# A Text Book of Practical Therapeutics, with Especial Reference to the Application of Remedial Measures to Disease and Their Employment upon a Rational Basis

**Published:** 1890-12

**Authors:** Hugh Hagan


A TEXT BOOK OF PRACTICAL THERAPEUTICS, WITH ESPECIAL REFERENCE TO THE APPLICATION OF REMEDIAL MEASURES TO DISEASE AND THEIR EMPLOYMENT UPON A RATIONAL BASIS. By Hobart Amory Hare, M. D. (University of Pennsylvania), B. Sc. Published by Lea Brothers & Co., Philadelphia. 1890.

This book will be found to fill a long felt need of the profession, for the busy practitioner is ever in need of a reference book, in which he may find practical hints on which he may rely. Dr. Hare has divided the work into parts, all of which are eminently instructive and comprehensive.

Part II. is especially valuable on account of the succinct description of the drugs in common use, especially of those new drugs which as yet have not been made officinal. Here the student finds, alphabetically arranged, all of those new drugs which the profession have lately tried, fully described as to their chemistry, physiological action and therapeutical uses. In no other work that I have seen have these drugs been sufficiently noted or classified. This feature alone will suffice to secure the general use of the work.

Parts III. and IV. are well worthy a place on the library shelf, and though not as much needed as part II., are well worth the author's effort. HUGH HAGAN.

